# Drug-Target Interaction Prediction Based on Drug Fingerprint Information and Protein Sequence

**DOI:** 10.3390/molecules24162999

**Published:** 2019-08-19

**Authors:** Yang Li, Yu-An Huang, Zhu-Hong You, Li-Ping Li, Zheng Wang

**Affiliations:** School of Information Engineering, Xijing University, Xi’an 710123, China

**Keywords:** drug-target interactions, local phase quantization, rotation forest, drug substructure fingerprint

## Abstract

The identification of drug-target interactions (DTIs) is a critical step in drug development. Experimental methods that are based on clinical trials to discover DTIs are time-consuming, expensive, and challenging. Therefore, as complementary to it, developing new computational methods for predicting novel DTI is of great significance with regards to saving cost and shortening the development period. In this paper, we present a novel computational model for predicting DTIs, which uses the sequence information of proteins and a rotation forest classifier. Specifically, all of the target protein sequences are first converted to a position-specific scoring matrix (PSSM) to retain evolutionary information. We then use local phase quantization (LPQ) descriptors to extract evolutionary information in the PSSM. On the other hand, substructure fingerprint information is utilized to extract the features of the drug. We finally combine the features of drugs and protein together to represent features of each drug-target pair and use a rotation forest classifier to calculate the scores of interaction possibility, for a global DTI prediction. The experimental results indicate that the proposed model is effective, achieving average accuracies of 89.15%, 86.01%, 82.20%, and 71.67% on four datasets (i.e., enzyme, ion channel, G protein-coupled receptors (GPCR), and nuclear receptor), respectively. In addition, we compared the prediction performance of the rotation forest classifier with another popular classifier, support vector machine, on the same dataset. Several types of methods previously proposed are also implemented on the same datasets for performance comparison. The comparison results demonstrate the superiority of the proposed method to the others. We anticipate that the proposed method can be used as an effective tool for predicting drug-target interactions on a large scale, given the information of protein sequences and drug fingerprints.

## 1. Introduction

Identifying drug-target interactions (DTI) plays a pivotal role in drug discovery. Drugs usually interact with one or more proteins to achieve their functions. However, discovering novel interactions between drugs and target proteins is crucial for the development of new drugs, since the aberrant expression of proteins may cause side effects of drugs [1]. In the past decades, researchers have been identifying drug-target interactions through clinical observation and biological experiments. However, using these experimentally based methods is still time-consuming and expensive. In addition, they also face the problem of high attrition rate [2]. Therefore, the use of computational techniques to predict drug-target interactions has become a hot research topic in the field of molecular pharmacology in recent years. The US Food and Drug Administration (FDA) report that the development of a new type of drug can at least cost billions of dollars. The rate for drugs to be approved for marketing is still very low in recent years due to their uncertain side effects. It is an urgent need to develop new methods that can predict drug-target interactions on a large scale to reduce the development period and cost of drug discovery [3].

In recent years, more and more drug-target interactions have been discovered by clinical research and stored in some public databases, offering data resource for computation method to train reliable prediction model for DTIs. Different types of databases that are used for drug-target relationships have been established and public released, such as SuperTarget and Matador [4], DrugBank [5], Therapeutic Target Database (TTD) [6], and Kyoto Encyclopedia of Genes and Genomes (KEGG) [7], providing data resources for computational tools.

To date, traditional computational methods for predicting drug-target interactions include docking simulations [8], ligand-based methods [9], and literature text mining methods [10]. However, these three types of methods still have some limitations. The docking simulation method requires usable three-dimensional (3D) structural information of the target protein, which is only available for a small fraction of proteins. Therefore, it fails to be applied to predict DTIs on a large scale. Ligand-based methods typically do not perform well with target proteins due to the limited number of known ligands. The text mining method mainly relies on keywords to search, so it is difficult to detect novel interactions between the drugs and target proteins. However, protein meta-structure is a new approach for chemical and molecular biology. It effectively identifies possible chemical fragments, which can also be used for fragment-based drug design. This method is solely based on primary sequence information and it does not require 3D protein structure information, so it allows a wider application for predicting drug-target interactions [11].

In recent years, researchers have developed different types of computational methods for inferring potential drug-target interactions. For example, Chen et al. [12] proposed a novel computational model combining a machine learning-based method and a network-based method. Wu et al. [13] proposed a useful tool, called substructure-drug-target network-based inference (SDTNBI), to identify potential drug-target interactions. The model combines topologic information of the DTI network and chemoinformatics features to implement repositioning predictions on four benchmark datasets: kinases, GPCRs, ion channels, and nuclear receptors. Mei et al. [14] proposed an effective algorithm, called BLM-NII, to identify drug-target interactions, which combines the Neighbor-based Interaction-profile Inferring method and the Bipartite Local Model. Yamanishi et al. [15] developed a supervised method that was based on a bipartite graph framework to predict unknown drug-target interactions. Specifically, the method maps geometric space and chemical space into a unified space called the pharmacological space. Xia et al. [16] developed a regularized semi-supervised learning method (NetLapRLS), which predicts DTI based on the genomic space, chemical space, and drug-protein interaction network space. Cheng et al. [17] developed three types of methods that were based on complex network theory to identify interactions between potential drugs and targets, which are network-based inference (NBI), target-based similarity inference (TBSI), and drug-based similarity inference (DBSI). Kuang et al. [18] proposed an efficient method that is based on the technique of eigenvalue transformation, combining a semi-supervised link prediction classifier (SLP) and a regularized least squares classifier (RLS) to predict drug-target interactions. More recently, Wang et al. [19] presented a computational approach to infer potential drug-target interactions. Specifically, the method converts the protein sequence into a position-specific scoring matrix (PSSM) while using biological evolutionary information and encodes the drug molecule as a fingerprint feature vector. Based on such feature information, feature extraction is performed on PSSM by using the auto-covariance (AC) algorithm.

In this work, we present a novel computational approach only using the information of target protein sequences and drug substructure fingerprints to predict drug-target interactions on a large scale. It can generally be divided into three steps: first, we convert all the target protein sequences into PSSM, considering the biological evolutionary information between different types of amino acids. Meanwhile, molecular substructure fingerprints are used as the features of drugs. Second, an efficient feature extraction method that is based on local phase quantization (LPQ) is used to convert the PSSMs into vectors. Third, an ensemble classifier, rotation forest, is adopted to perform DTI predictions on four gold standard datasets including enzymes, ion channels, GPCRs, and nuclear receptors. We also compare the proposed method with several types of existing methods to evaluate the prediction performance. The experimental results further indicate that the proposed method can effectively predict drug-target interactions.

## 2. Results and Discussion

### 2.1. Evaluation Criteria

In this paper, we used four evaluation criteria to evaluate the prediction performance of the proposed method on four golden standard datasets, including accuracy (Acc.), sensitivity (Sen.), precision (Pre.), and Matthews correlation coefficient (MCC). They can be formulated, as follows:

(1) $$Acc.=\frac{TN+TP}{TN+FN+TP+FP}$$

(2) $$Sen.=\frac{TP}{FN+TP}$$

(3) $$Pre.=\frac{TP}{FP+TP}$$

(4) $$MCC=\frac{TN\times TP-FN\times FP}{\sqrt{\left(TN+FN)\times (TP+FP)\times (TN+FP)\times (TP+FN\right)}}.$$

where true negative (TN) is the number of true samples predicted to have non-interacting drug-target pairs; true positive (TP) is the count of true samples predicted to have interacting drug-target pairs; false negative (FN) is the count of interacting drug-target pairs that are predicted to have no interaction; false positives (FP) refers to the number of non-interacting drug-target pairs that are predicted to interact. We adopted the receiver operating characteristic (ROC) [20] curves and then calculated the area under the curve (AUC) to quantitively evaluate the prediction performance of the proposed model.

### 2.2. Performance Evaluations on Four Golden Standard Datasets

Five-fold cross-validation method was applied to predict drug-target interactions on four datasets (i.e., enzymes, ion channels, GPCRs, and nuclear receptors) in this experiment to further evaluate the reliability of the proposed method. Specifically, we partitioned the datasets into five roughly equal parts, four of which were used to construct a training set and the rest one was used as a testing set. In this way, we can obtain five models by training it on five training datasets. Finally, the feature vector of each sample in testing datasets was used as the inputs of the trained prediction model and obtain a prediction score, which measures the possibility for a given drug-target pair to be interactive. We also implemented a series of experiments to optimize the parameters of the rotation forest classifier for better prediction performance. As a result, the parameter *K* and *L* are set to be 22 and 25, respectively, and, for the sake of fairness, they would be kept the same for all of the experiments in this work. Here, *K* represents the number of feature subsets and *L* represents the number of decision trees. Table 1, Table 2, Table 3 and Table 4 present the DTI prediction results for the proposed model while using the five-fold cross-validation method on the gold standard datasets (including enzymes, ion channels, GPCRs, and nuclear receptors).

**Figure 1:**
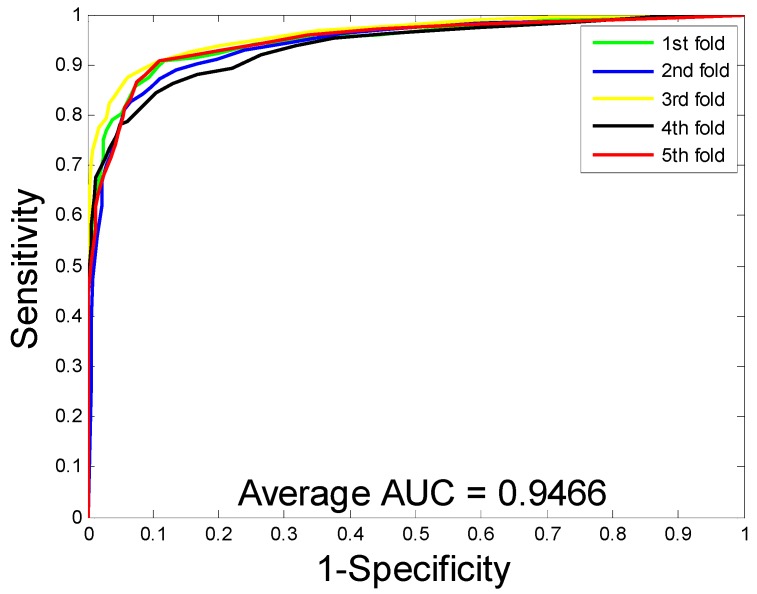
The receiver operating characteristic (ROC) curves are generated by our method on enzyme dataset.

**Figure 2:**
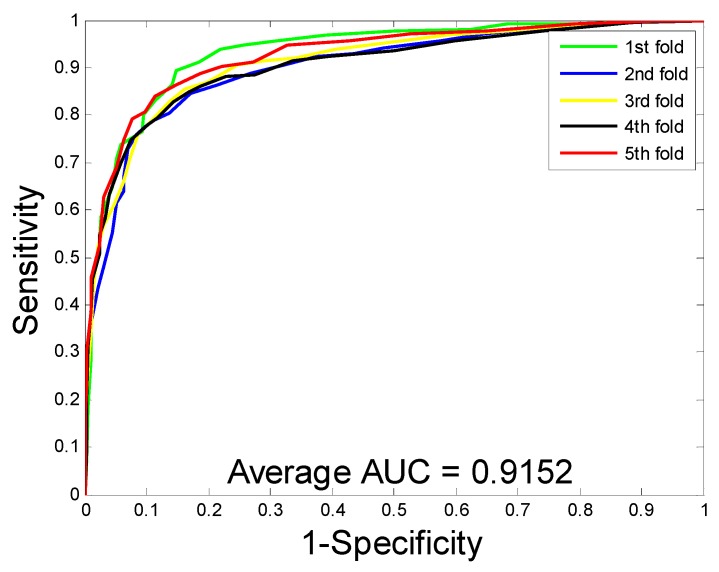
The ROC curves are generated by our method on ion channel dataset.

**Figure 3:**
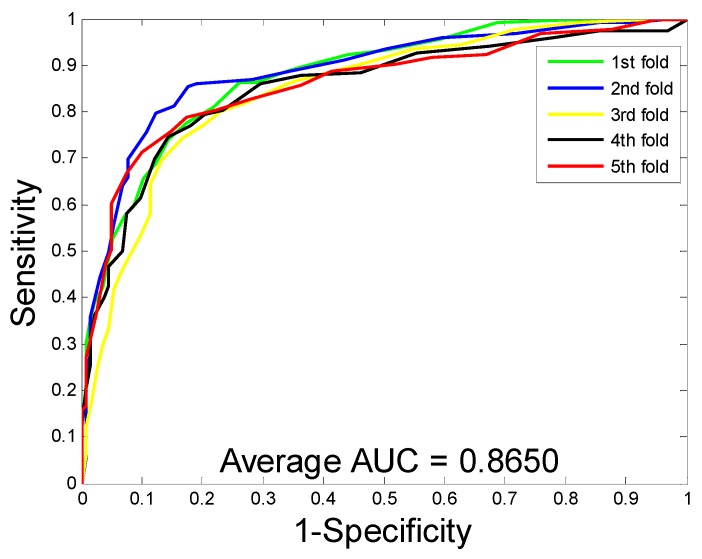
The ROC curves are generated by our method on GPCR dataset.

**Figure 4:**
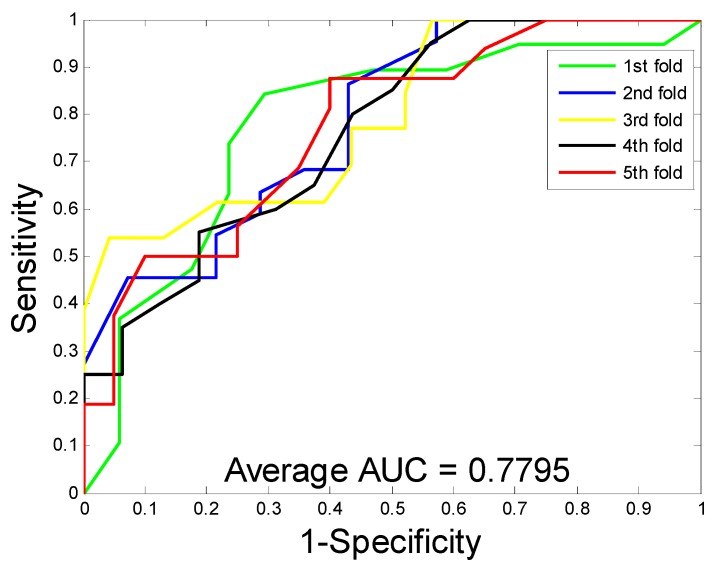
The ROC curves are generated by our method on nuclear receptor dataset.

When predicting DTI on the enzyme dataset, the average accuracy, precision, sensitivity, MCC, and AUC were 89.15%, 91.06%, 86.85%, 80.65%, and 94.66%, with corresponding standard deviations of 1.36%, 1.83%, 1.34%, 2.10%, and 0.99%, respectively. With regards to the ion channel dataset, the average accuracy, precision, sensitivity, MCC, and AUC were 86.01%, 85.66%, 86.62%, 75.94%, and 91.52%, with corresponding standard deviations of 1.61%, 3.23%, 3.10%, 2.33%, and 1.36%, respectively. When performing DTI prediction on the GPCR dataset, the average accuracy, precision, sensitivity, MCC, and AUC were 82.20%, 82.83%, 81.28%, 70.62%, and 86.50% with corresponding standard deviations of 1.19%, 2.24%, 2.75%, 1.65%, and 1.68%, respectively. When predicting DTI on the nuclear receptor dataset, the average accuracy, precision, sensitivity, MCC, and AUC were 71.67%, 69.61%, 76.45%, 57.97%, and 77.95%, with corresponding standard deviations of 3.62%, 10.43%, 10.61%, 2.00%, and 1.10%, respectively. We further calculate the corresponding TPRs and FPRs and plot the ROC curves, which is as shown in Figure 1, Figure 2, Figure 3 and Figure 4, respectively.

### 2.3. Comparison with Support Vector Machine Classifier

**Figure 5:**
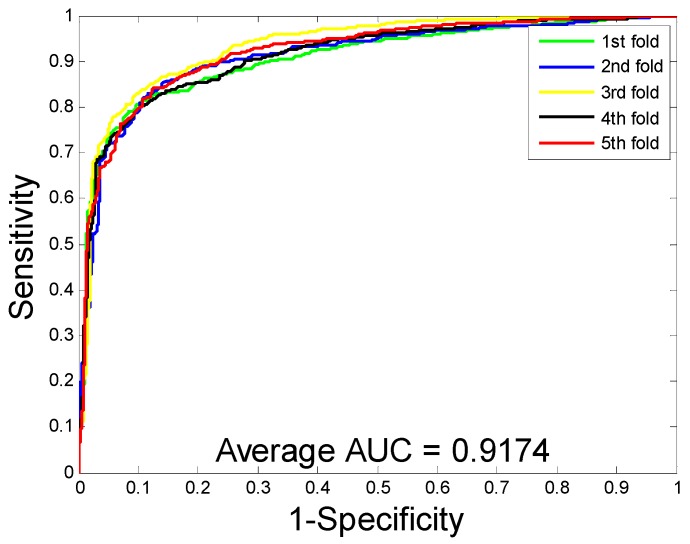
The ROC curves are generated by the support vector machine (SVM) classifier on enzyme dataset.

We combined the same feature extraction method with the popular support vector machine (SVM) classifier to predict the DTIs on the enzyme dataset to further assess the prediction results of the proposed method. Here, the five-fold cross-validation method is used in this experiment to compare the prediction performance of the SVM with that of the proposed model. The LIBSVM tool [21] is applied to perform classification for drug-target interaction prediction. At the same time, we also need to use the grid search method to optimize the relevant parameters of the SVM classifier in the experiment. Table 5 summarizes the predicted results of the RF and SVM classifiers on the enzyme dataset. As a result, the average values of accuracy, precision, sensitivity, and MCC were 85.20%, 85.73%, 84.45%, and 74.76%, respectively, with standard deviations of 0.82%, 0.99%, 1.64%, and 1.15%, respectively. Figure 5 shows the ROC curves that were generated on the enzyme dataset while using SVM. Comparing the prediction results of the two classifiers, we can see that, while using the same feature descriptor, the prediction performance that was yielded by the rotation forest classifier is significantly better than that yielded by the support vector machine classifier.

### 2.4. Comparison with Other Methods

To date, different types of computational models have been developed for detecting drug-target interactions. In this section, we compare the proposed model with the other four existing models, including DBSI [17], KBMF2K [22], NetCBP [23], and Yamanishi [15] on the golden standard datasets to better evaluate the prediction performance of the proposed method. Here, we explored five different methods for drug-target interaction prediction on the four datasets and calculated the corresponding AUC values. Table 6 lists the comparison results of these models. It can be seen that the proposed method achieves AUC values of 0.9466, 0.9152, 0.8650, and 0.7795 on the enzyme, ion channel, GPCR, and nuclear receptor datasets, respectively. Although the proposed method AUC value is 0.0599 lower than the NetCBP method on the nuclear receptor dataset, our method obtained an average AUC increase of 0.1146, 0.1118, and 0.0080 on the enzyme, ion channel, and GPCR datasets, respectively. The comparison results show that the proposed model is effectively improved in the predictive performance of drug-target interactions.

## 3. Materials and Methods

### 3.1. Golden Standard Datasets

In this article, we explore four golden standard datasets to evaluate the prediction performance of the proposed with regards to its prediction on drug-target interactions. These datasets are collected from four databases, KEGG BRITE [7], SuperTarget [4], BRENDA [24], and DrugBank [5]. In each dataset, the data cover four types of drug target families, namely enzymes, ion channels, GPCRs, and nuclear receptors. The numbers of known drug targeting enzymes, ion channels, GPCRs, and nuclear receptors are 445, 210, 223, and 54, respectively, and the numbers of their corresponding target proteins are 664, 204, 95, and 26, respectively. The total number of DTIs in these datasets was 5127. Among them, the numbers of known DITs for the enzyme, ion channel, GPCR, and nuclear receptor datasets are 2926, 1476, 635, and 90, respectively. Table 7 shows the statistical information in different datasets.

In this work, we represent the network of drug-target interactions as a bipartite graph, in which the nodes refer to target proteins or drug molecules, and the links are the interactions between them. The network is sparse, as the number of the known DTIs is limited. There are totally 295,480 (445 × 664) connections in the corresponding bipartite graph. But only 2926 edges existing and represented as the known drug-target interactions. In this case, the number of possible negative samples is 292,554 (295480−2926), significantly larger than that of positive samples (2926). To deal with this problem that is caused by the sample unbalance, we randomly selected the negative sample from the unlabeled drug-protein pairs with the same number of positive samples. In general, the negative sample sets that were obtained in this way may contain a small number of really interacting drug-target pairs. However, when considering the large-scale study of DTIs, the number of real interaction pairs selected from the negative sets is quite small. As a result, the number of enzyme, ion channel, GPCR, and nuclear receptor datasets in the negative samples was 2926, 1476, 635, and 90, respectively.

### 3.2. Drug Substructure Feature

In previous studies, feature information for drugs include topological, geometrical, constitutional, and quantum chemical properties. In this work, we use molecular fingerprints as the drug feature information to consider the substructures of drug compounds. Each bit in the binary fingerprint vectors is used to represent a specific substructure of a certain molecule [25]. Substructure fingerprints can directly encode structural information for a given drug compound into a series of binary bits, indicating the presence of a specific substructure of the drug molecule. There is a list of SMARTS substructure patterns in the predefined dictionary. Based on the predefined SMARTS pattern, the corresponding bit in the fingerprint vector is set to 1 if a given drug molecule contains its corresponding substructure, and otherwise it is assigned to be 0. In this study, we selected the chemical structure of the molecular substructure fingerprints that were collected from the PubChem system (available at https://pubchem.ncbi.nlm.nih.gov/). As a result, the drug molecule feature is a binary vector of 881dimensions.

### 3.3. Position-Specific Scoring Matrix

There are currently many effective ways to convert protein sequences into multidimensional feature vectors. For instance, by using statistical distributions of amino acids [26,27,28] or by using the physico-chemical properties of amino acids [29,30,31]. These methods provide a powerful basis for predicting drug-target interactions. Position-specific scoring matrix (PSSM) is widely used in previous research, including protein secondary structure prediction [32], protein binding site prediction [33], and prediction of disordered regions [34]. PSSM is also able to extract evolutionary information of 20 types of amino acids. Effective protein descriptors are crucial for the prediction of drug-target interactions. In this work, we adopted PSSM to extract target protein features for DTI prediction. For a given protein sequence, we converted it to PSSM while using a Position-specific iterated basic local alignment search tool (PSI-BLAST) [35]. The PSSM of the protein sequence can be expressed as:

(5) $$PSSM=(P_{1},P_{2},\ldots ,P_{i},\ldots ,P_{20})$$

where $P_{m}={\left(P_{1,m},P_{2,m},\ldots ,P_{T,m}\right)}^{T},(m=1,2,\ldots ,20)$; T is the length of an amino acid sequence. Finally, a matrix of T×20 can be constructed for each protein sequence. We set the relevant parameters of PSI-BLAST (E-value) as 0.001, the number of iterations as 3, and other parameters as default values in order to obtain highly homologous sequences. Details of PSI-BLAST can be accessed at https://blast.ncbi.nlm.nih.gov/Blast.cgi.

### 3.4. Local Phase Quantization

With the development of image processing techniques, many methods have emerged for extracting features from the data matrixes of original images. For example, local phase quantization (LPQ) was proposed as an effective operator for texture descriptors that were first proposed by Ojansivu et al. [36]. Specifically, LPQ remains the blur invariance property of the Fourier phase spectrum in the image matrixes, extracting local phase information based on two-dimensional (2-D) short-term Fourier transform (STFT) [37]. For a given original image f(x), its spatially invariant blurring in the observed image g(x) can be represented by convolution:

(6) g(x)=(f∗h)(x),

where h(x) is the point spread function (PSF) for the blur, ∗ denotes a two-dimensional convolution, and x represents a vector of coordinates ${\left[x,y\right]}^{T}$ in the image. In the Fourier domain, this can be expressed as:

(7) G(u)=F(u)·H(u),

where F(u), H(u), and G(u) are the discrete Fourier transform (DFT) functions of f(x), h(x), and g(x), respectively. Here, u denotes a vector of coordinates ${\left[u,v\right]}^{T}.$ Based on the characteristics of the Fourier transform, we can express the magnitude and phase as:

(8) |G(u)|=|F(u)|·|H(u)|, ∠G(u)=∠F(u)+∠H(u).

Suppose the blur h(x) is centrally symmetric, meaning that h(x)=h(−x), in which case its Fourier transform is always real-valued, so its phase can only be represented as a two-valued function. That is:

(9) $$∠H(u)=\{\begin{array}{c} 0\\ \pi \end{array}\begin{array}{cc} & \mathrm{if}\;H(u)\geq 0,\\ & \mathrm{if}\;H(u)<0. \end{array}$$

For all H(u)≥0, there is:

(10) ∠G(u)=∠F(u).

In the LPQ method, the shape of a regular PSF h(x) is usually similar to a sin or Gaussian function. It should be noted that the low frequency values of H(u) are positive. It uses two-dimensional DFT to extract local phase information in order to obtain local information efficiently. That is, the phase information is obtained by the rectangular M×M neighborhood $N_{x}$ at each pixel position x of a given image f(x). These local spectra are calculated while using a STFT, which can be defined as:

(11) $$F(u,x)=\sum_{y\in N_{x}}f(x-y)e^{-j2\pi yu^{T}}=w_{u}^{T}f_{x},$$

where $w_{u}$ denotes the basis vector of the two-dimensional DFT at frequency u and $f_{x}$ represents another vector containing all the $M^{2}$ image sample pixels from the neighborhood $N_{x}$. In order to improve the efficiency of the calculation, according to the separability of the basis functions, we can use the one-dimensional convolution formula for the rows and columns to calculate the STFT of each pixel position in the image. In the LPQ method, the calculation formulas of the local Fourier coefficients at four frequency points are: $u_{1}={\left[\alpha ,0\right]}^{T}$, $u_{2}={\left[0,\alpha \right]}^{T}$, $u_{3}={\left[\alpha ,\alpha \right]}^{T}$, and $u_{4}={\left[\alpha ,-\alpha \right]}^{T}$. Here, α is a sufficiently small frequency parameter. Thus, each pixel position can be represented by a vector.

(12) $$F_{x}^{c}=[F(u_{1},x),F(u_{2},x),F(u_{3},x),F(u_{4},x)],$$

(13) Fx=[Re{Fxc},Im{Fxc}]T,

where Re and Im represent the real and imaginary parts of a complex number, respectively. Next, we can use a simple scalar quantizer to calculate the phase information, as follows:

(14) $$q_{j}(x)=\{\begin{array}{c} 1\\ 0 \end{array}\begin{array}{cc} & \mathrm{if}\;g_{j}(x)\geq 0\\ & \mathrm{otherwise}\; \end{array},$$

where $g_{j}(x)$ refers to the *j*th component of the vector Gx=[Re{Fx},Im{Fx}]. After quantization, the quantized coefficients can be represented as the integer values between 0–255 by employing binary coding

(15) $$f_{\mathrm{LPQ}}(x)=\sum_{j=1}^{8}q_{j}(x)2^{j-1}.$$

**Figure 6:**
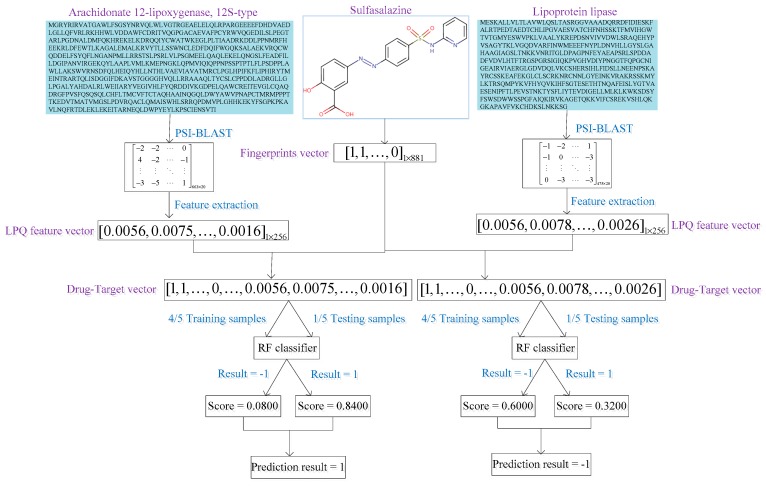
Flow chart for a given drug and target protein based on the proposed method.

As a result, we obtain the distribution of the integer values of all the pixels in the image f(x), and these results are used as a 256-dimensional feature vector for further classification. In this work, we used the LPQ method to analyze the four target protein datasets, and finally converted the PSSM of each protein sequence into a 256-dimensional feature vector. The predicted results for a given drug and target protein based on the proposed method are displayed in Figure 6. We illustrate the prediction of the interactions between a drug, Sulfasalazine, and two target protein sequences. The length of the sequence, named Arachidonate 12-lipoxygenase, 12*S*-type is 663, and the length of the sequence named Lipoprotein lipase is 475. According to the results, we can see that the drug Sulfasalazine is predicted to interact with target protein Arachidonate 12-lipoxygenase, 12*S*-type with possibility score of 0.844, and not to interact with target protein Lipoprotein lipase with possibility score of 0.3200.

### 3.5. Rotation Forest

Rotation Forest (RF) is a classification method that is widely used for supervised learning. RF was originally proposed by Rodriguez et al. [38], and it has outstanding prediction performance as an ensemble learning classifier. In the rotation forest algorithm, the feature set S is randomly divided into K subsets (K is a parameter in RF), and the bootstrap sampling technique is then applied to 75% of the original training samples on each feature subset to obtain a sparse rotation matrix. Next, the classifier is constructed by using the repeated projection features of the matrix multiple times, and the final class of the test sample is given in combination with the prediction result of the multiple classifiers.

Let X be the training sample set, which is a matrix of N×n, and it is composed of n feature vectors for each training sample N. Let the feature set be S and the corresponding class label be Y, denoted as ${\left(y_{1},y_{2},\ldots ,y_{n}\right)}^{T}$. Suppose that there are a total of L decision trees in a rotation forest classifier, which are denoted as $D_{1},\ldots ,D_{L}$, respectively. Subsequently, for an individual classifier $D_{i}$, the implementation of the training set is as follows:

(I) The feature set S is randomly divided into K disjoint parts, and the feature number of each subset is C=n/K.

(II) Let $S_{ij}$ denote the *j*th subset of features, which is the training set for classifier $D_{i}$. Afterwards, for each such subset, we use a bootstrap sampling technique to reconstruct a new training set ${X^{\prime }}_{ij}$ from 75 percent of the original training dataset X.

(III) Apply principal component analysis to ${X^{\prime }}_{ij}$ using only the C features in $S_{ij}$. The coefficients of the principal components are stored in a matrix $M_{ij}$ and their size is C×1, which are denoted as $a_{{}_{ij}}^{\left(1\right)},\ldots ,a_{{}_{ij}}^{\left(C_{j}\right)}$.

(IV) The coefficients obtained in the matrix $M_{ij}$ are arranged into a sparse rotation matrix $G_{i}$, as follows:

(16) $$G_{i}=\left[\begin{array}{cccc} a_{i1}^{\left(1\right)},\ldots ,a_{i1}^{\left(C_{1}\right)} & \left\{0\right\} & \cdots & \left\{0\right\}\\ \left\{0\right\} & a_{i2}^{\left(1\right)},\ldots ,a_{i2}^{\left(C_{2}\right)} & \cdots & \left\{0\right\}\\ \vdots & \vdots & \ddots & \vdots \\ \left\{0\right\} & \left\{0\right\} & \cdots & a_{iK}^{\left(1\right)},\ldots ,a_{iK}^{\left(C_{K}\right)} \end{array}\right].$$

For a given test sample x during the classification period, assuming $d_{ij}(xG_{i}^{a})$ be the probability that is obtained by the classifier $D_{i}$, which is used to discriminate that x belongs to class $y_{i}$. Next, calculate the confidence of the class by the average combination method, the formula is as follows:

(17) $$\mu_{j}(x)=\frac{1}{L}\sum_{i=1}^{L}d_{ij}(xG_{i}^{a}).$$

Finally, x is assigned to a class with the largest calculation result.

## 4. Conclusions

When considering the drug substructure fingerprints, target protein sequences, and known drug-target interactions as important information for DTI prediction, here we developed a novel computational method for predicting DTIs on a large scale. Specifically, the proposed method combines position-specific scoring matrix (PSSM), local phase quantization (LPQ), and rotation forest (RF) classifier to predict DTIs. The five-fold cross-validation method was used in this work to assess the predictive performance of the proposed method on the golden standard datasets. As a result, the average accuracy that was yielded by our method achieved 89.15%, 86.01%, 82.20%, and 71.67% on enzymes, ion channels, GPCRs, and nuclear receptors datasets, respectively. To better illustrate the predictive power of the proposed method, we also compared it to the support vector machine classifier as well as some other previous methods on the golden standard datasets. The experimental results further indicate that the proposed method can effectively predict drug-target interactions. We anticipate that the proposed model can serve as a useful tool for predicting DTIs on a large scale in the future research of molecular pharmacology.

## Figures and Tables

**Table 1:** The five-fold cross-validation results achieved on enzyme dataset by using the proposed model.

Test Set	Acc.(%)	Pre.(%)	Sen.(%)	MCC(%)	AUC(%)
1	89.74	90.68	87.79	81.54	94.77
2	88.55	89.61	87.81	79.71	94.04
3	90.60	93.73	87.46	82.94	96.16
4	87.09	89.30	84.63	77.50	93.55
5	89.76	92.01	86.54	81.55	94.82
**Average**	89.15 ± 1.36	91.06 ± 1.83	86.85 ± 1.34	80.65 ± 2.10	94.66 ± 0.99

**Table 2:** The five-fold cross-validation results achieved on ion channel dataset by using the proposed model.

Test Set	Acc.(%)	Pre.(%)	Sen.(%)	MCC(%)	AUC(%)
1	87.97	86.03	90.94	78.78	93.27
2	84.41	84.59	85.15	73.65	90.16
3	85.25	81.46	88.81	74.80	91.14
4	84.92	85.81	83.78	74.38	90.44
5	87.50	90.43	84.44	78.10	92.62
**Average**	86.01 ± 1.61	85.66 ± 3.23	86.62 ± 3.10	75.94 ± 2.33	91.52 ± 1.36

**Table 3:** The five-fold cross-validation results achieved on GPCR dataset by using the proposed model.

Test Set	Acc.(%)	Pre.(%)	Sen.(%)	MCC(%)	AUC(%)
1	82.68	79.51	83.62	71.23	87.83
2	83.46	82.40	83.74	72.38	88.66
3	80.31	82.73	81.56	68.04	84.77
4	81.89	83.93	77.05	70.11	85.21
5	82.68	85.60	80.45	71.32	86.01
**Average**	82.20 ± 1.19	82.83 ± 2.24	81.28 ± 2.75	70.62 ± 1.65	86.50 ± 1.68

**Table 4:** The five-fold cross-validation results achieved on nuclear receptor dataset by using the proposed model.

Test Set	Acc.(%)	Pre.(%)	Sen.(%)	MCC(%)	AUC(%)
1	72.22	80.00	63.16	59.25	77.86
2	77.78	76.92	90.91	60.78	78.57
3	69.44	55.56	76.92	55.90	79.43
4	69.44	73.68	70.00	57.23	76.56
5	69.44	61.90	81.25	56.69	77.34
**Average**	71.67 ± 3.62	69.61 ± 10.43	76.45 ± 10.61	57.97 ± 2.00	77.95 ± 1.10

**Table 5:** The five-fold cross-validation results achieved on the enzyme dataset by using the rotation forest classifier and the support vector machine classifier.

Test Set	Acc.(%)	Pre.(%)	Sen.(%)	MCC(%)
PSSM+LPQ+RF				
1	89.74	90.68	87.79	81.54
2	88.55	89.61	87.81	79.71
3	90.60	93.73	87.46	82.94
4	87.09	89.30	84.63	77.50
5	89.76	92.01	86.54	81.55
**Average**	89.15 ± 1.36	91.06 ± 1.83	86.85 ± 1.34	80.65 ± 2.10
PSSM+LPQ+SVM				
1	85.04	86.25	82.12	74.46
2	85.47	86.05	85.48	75.15
3	85.90	86.02	86.45	75.76
4	83.85	83.98	84.12	72.91
5	85.75	86.36	84.09	75.53
**Average**	85.20 ± 0.82	85.73 ± 0.99	84.45 ± 1.64	74.76 ± 1.15

**Table 6:** The comparison of the area under the curve (AUC) values obtained between the proposed method and other existing methods on the gold standard datasets.

Dataset	Our Method	DBSI	KBMF2K	NetCBP	Yamanishi
Enzymes	0.9466	0.8075	0.832	0.8251	0.821
Ion Channels	0.9152	0.8029	0.799	0.8034	0.692
GPCRs	0.8650	0.8022	0.857	0.8235	0.811
Nuclear Receptors	0.7795	0.7578	0.824	0.8394	0.814

**Table 7:** The number of four drug-target interaction datasets.

Dataset	Drug Compounds	Target Proteins	Interactions
Enzyme	445	664	2926
Ion channel	210	204	1476
GPCR	223	95	635
Nuclear receptor	54	26	90
